# A functional tandem-repeats polymorphism in the downstream of *TERT *is associated with the risk of nasopharyngeal carcinoma in Chinese population

**DOI:** 10.1186/1741-7015-9-106

**Published:** 2011-09-20

**Authors:** Yang Zhang, Hongxing Zhang, Yun Zhai, Zhifu Wang, Fuchao Ma, Hongxue Wang, Peiyao Li, Ying Zhang, Lixia Yu, Ying Cui, Fuchu He, Gangqiao Zhou

**Affiliations:** 1 Institute of Basic Medical Sciences, Chinese Academy of Medical Sciences & Peking Union Medical College, Beijing, China; 2 State Key Laboratory of Proteomics, Beijing Proteome Research Center, Beijing Institute of Radiation Medicine, Beijing, China; 3 Affiliated Cancer Hospital of Guangxi Medical University, Nanning, Guangxi, China

## Abstract

**Background:**

Increases in human telomerase reverse transcriptase (TERT) expression and telomerase activity are frequently seen in nasopharyngeal carcinoma (NPC). Recently, a variable tandem-repeats polymorphism, MNS16A, located in the downstream region of the *TERT *gene, was identified and reported to have an effect on *TERT *expression and telomerase activity. We examined whether the functional MNS16A was related to the risk of occurrence or progression of NPC in the Chinese population.

**Methods:**

We genotyped the MNS16A polymorphism in a case-control study of 855 patients with NPC and 1036 cancer-free controls using PCR, and determined genotype by classifying the DNA band of 243 or 272 base pairs (bp) as the short (*S*) allele and 302 or 333 bp as the long (*L*) allele. The genetic associations with the risk of NPC were analyzed by logistic regression.

**Results:**

The MNS16A genotype was not associated with the progression of NPC. However, individuals carrying the *S *alleles (*SL *+ *SS *genotype) had a significantly reduced risk of NPC occurrence compared with those carrying the *LL *genotype (odds ratio (OR) = 0. 71, 95% confidence interval (CI) = 0. 52 to 0. 96, *P *= 0. 025). Using a immunohistochemical assay on the NPC tissues, the *SL *genotype carriers were found to have lower TERT expression than the *LL *genotype carriers (*P *= 0. 035).

**Conclusions:**

Our study indicates that the *TERT *MNS16A polymorphism may contribute to the risk of NPC onset in Chinese population.

## Background

Nasopharyngeal carcinoma (NPC) is an epithelial malignancy with strikingly variable racial and geographic distribution. It is particularly prevalent in populations from southeast Asia, with an incidence rate ranging from 15 to 50 per 100,000, which is ~100 times higher than that in Caucasian populations [1]. Like other kinds of cancer, the risk of NPC is influenced by both genetic and environmental risk factors. Epstein-Barr virus (EBV) infection has a crucial role in the pathogenesis of NPC. Numerous environmental risk factors, including long-term cigarette smoking, occupational formaldehyde exposure and various dietary factors, have been reported to confer a risk of developing NPC [2]. In addition, numerous genetic linkage and association studies have reported a few genes contributing to the risk of this malignancy [3-6]. The identification of susceptibility genes contributing to NPC may help to clarify the pathogenesis of carcinogenesis, and improve the prevention and treatment of this malignancy.

Telomeres are structures that cap the distal ends of chromosomes, and function to prevent chromosome degradation, end-to-end fusions, rearrangements and chromosome attrition [7]. Telomere-driven genome instability is a widespread cause of genome instability in cancer, and is thought to be the crucial event in tumorigenesis [7]. Telomerase is a reverse transcriptase enzyme that can elongate the TTAGGG repeats of telomeres in cells, in which it is expressed to maintain the ends of chromosomes and sustain cellular immortality. Telomerase is a holoenzyme, and its catalytic subunit (telomerase reverse transcriptase, *TERT*) is the core component responsible for its enzymatic activity [8]. In humans, telomerase activity is undetectable in most normal cells, with the exception of peripheral and cord blood, and bone marrow leukocytes [9]. The existence of detectable telomerase expression in almost all types of human cancers suggests that enhanced telomerase expression is crucial for cell growth during tumorigenesis [10-12].

With regard to NPC, increased telomerase activity and TERT expression is also common in tumor tissues, and can be detected at high frequencies compared with normal nasopharyngeal epithelium and tissues [12,13]. It has been shown that normal nasopharyngeal epithelial cells can be transformed into immortalized cells by activating telomerase [14]. By contrast, small hairpin (sh)RNA directed against TERT inhibits cell viability by regulating telomerase activity and its related protein expression in NPC cells [15]. Additionally, latent membrane protein (LMP)1, which is the principal oncoprotein encoded by EBV, was able to induce the direct binding of TERT to nuclear factor kappa B (NF-κB) p65 in NPC cells, then lead to translocation of both proteins from the cytoplasm to the nucleus, and subsequent activation of telomerase and cell immortalization [16]. Another study found that small interfering (si)RNA targeting LMP1 can induce apoptosis in EBV-positive lymphoma cells, and is associated with inhibition of telomerase activity and expression [17]. Furthermore, with regard to the clinicopathological features in patients with NPC, telomerase activity was seen more frequently in the advanced clinical stages (III to IV) and in patients with lymph-node metastasis (N1 to N3) [18,19]. Taken together, these observations indicate that telomerase and *TERT *play an important role in the occurrence and progression of NPC [20].

Based on the functional relevance of TERT in the pathogenesis of NPC described above, we hypothesized that the *TERT *might be an excellent biological candidate susceptibility gene for NPC, and that functional polymorphisms within *TERT *might result in genotype-dependent differences in susceptibility to NPC.

In a previous study, a functional variable number of tandem repeats (VNTR) polymorphism, MNS16A, was identified in the downstream region of the *TERT *gene on chromosome 5p15. 33 [21]. The region containing MNS16A was found to have some promoter activity that was influenced by the length of the MNS16A tandem repeats, suggesting a potential role of this minisatellite in regulating both the expression of the antisense *TERT *mRNA level and the activity of telomerase [21]. Therefore, we hypothesized that this functional polymorphism in the downstream of *TERT *gene might contribute to the tumor susceptibility. Indeed, several genetic epidemiological studies have shown MNS16A to be a potential risk factor and/or a prognostic marker of lung [21-23], breast [24], brain [25-27] and colorectal [28] cancers. However, the role of MNS16A in NPC has never been specifically investigated. In this study, we examined whether the functional polymorphism in the downstream region of *TERT*, MNS16A, has any bearing on the occurrence or progression of NPC in the Chinese population.

## Methods

### Ethics

The study was approved by the ethics committee of Beijing Institute of Radiation Medicine (Beijing, China), and written informed consent was obtained from all the participants involved.

### Study population

This case-control study enrolled 855 patients with incident NPC and 1036 control subjects (see Additional file 1, Table S1). All subjects were unrelated ethnic Chinese adults, who were resident in Nanning City (Nanning, China) and the surrounding regions. At recruitment, personal information on demographic factors, medical history, and tobacco and alcohol use were collected via a structured questionnaire.

Diagnosis of patients and the inclusion and exclusion criteria for patients and controls have been described in detail previously [29]. Briefly, all patients were consecutively recruited between September 2003 and January 2008 at the Guangxi Cancer Hospital (Nanning, China) in Guangxi province, a well-known high-risk region for NPC located in southern China. The response rate for the patients was 95%.

The inclusion criterion for patients with NPC was that their diagnosis pf NPC had been histopathologically confirmed. Patients who had received chemotherapy or radiotherapy before undergoing surgery or had other type of cancer were excluded from this study. Tumor staging was performed according to the tumor, node, metastasis (TNM) classification of the 1997 American Joint Committee on Cancer (AJCC) system. All TNM classifications were determined by senior pathologists of the hospital, on the basis of the postoperative histopathologic examination. All the controls were recruited in the same regions during the same time that the NPC cases were collected. The selection criteria for the controls included no individual history of cancer, and frequency matching to the cases on gender and age (± 5 years). The response rate for the controls was 96%.

From the group of 855 patients with incident NPC, 41 patients who had undergone resection before receiving any further treatment at Guangxi Cancer Hospital were selected, and primary NPC biopsies were collected from them (see Additional file 1, Table S2). The histological type of all tumor tissues was poorly differentiated squamous cell carcinoma (SCC). Histological non-cancerous nasopharyngeal epithelium tissues were collected from 13 of the 1036 control subjects (see Additional file 1, Table S2). All the tissues were fixed in paraformaldehyde, embedded in paraffin wax, and prepared for immunohistochemical (IHC) staining.

### Polymorphism genotyping

The MNS16A polymorphism was genotyped by PCR with the primers described previously [21] (forward primer: 5'-AGGATTCTGATCTCTGAAGGGTG-3', located at nucleotide (nt) 22591; reverse primer: 5'-TCTGCCTGAGGAAGGACGTATG-3', located at nt 22871. GenBank: AF128894. 1). Briefly, PCR was performed in a total volume of 15 μl with an initial denaturing step for 5 minutes at 95°C, followed by 43 cycles of 30 seconds at 95°C, 45 seconds at 61°C and 1 minutes at 72°C, and a final extension step with 10 minutes at 72°C. The PCR products were separated in a 2. 5% agarose gel, visualized with UV light, and photographed. Four lengths of repeat polymorphisms were seen: 243, 272, 302 and 333 bp. As reported previously [24], we also saw one extra band for every heterozygous genotype, which resulted from a conformational change of the PCR products with different allele lengths. However, when the denatured PCR products from every heterozygous subject were separated in an 8% denatured polyacrylamide gel, the extra band disappeared (see Additional file 1, Figure S1).

For further quality control, a new set of primer pairs was designed to validate the above genotypes (forward primer: 5'-AAAGTCACATTCCGCCTCTG-3'' located at nt 22549; reverse primer:5'-TCTGCAATCCTTCCAGTTCC-3', located at nt 22894. GenBank: AF128894. 1). PCR was performed in a total volume of 15 μl with an initial denaturing step for 5 minutes at 95°C, followed by 35 cycles of 30 seconds at 95°C, 45 seconds at 52°C and 1 minute at 72°C, and a final extension step of 10 minutes at 72°C. Using the new primers, four lengths of repeats polymorphism were also seen: 306, 335, 365 and 396 bp, corresponding to the previously seen bands of 243, 272, 302 and 333 bp, respectively. One extra band for every heterozygous genotype was still visible in a 2. 5% agarose gel and disappeared in an 8% denatured polyacrylamide gel (see Additional file 1, Figure S2). A masked, random sample of 15% of the cases and controls was tested with the new primers, and all results had 100% concordance. Genotyping was performed by staff blinded to the subjects' status (case or control).

### IHC staining analysis

NPC tissues (n = 41) and non-tumor nasopharyngeal tissues (n = 13), which had been fixed with paraformaldehyde and embedded in paraffin wax, were analyzed for protein expression of TERT. Two slides from each biopsy were stained with hematoxylin and eosin for routine histological evaluation. The slides were washed in xylene to remove the paraffin wax, and then rehydrated through serial dilutions of alcohol, followed by washing in phosphate-buffered saline (PBS) pH 7. 2. All subsequent washes were buffered via the same protocol. The slides were then incubated with 3% H_2_O_2 _for 10 minutes to reduce non-specific staining. Treated slides were placed in citrate buffer pH 6. 0, and heated in a pressure cooker for 2 minutes. The slides were then incubated for overnight at 4°C with rabbit anti-human TERT monoclonal antiboby (LS-C49516; Lifespan, Seattle, WA and USA.). After washing, the slides were treated with a commercial kit (MaxVision™ HRP-Polymer Anti-Rabbit Immunohistochemistry Kit; Maxim Co., China). All slides were then incubated with 3,3-diaminobenzidine tetrahydrochloride (DAB), and the cells were counter-stained with hematoxylin. Negative and positive controls were performed at the same time. The slides were mounted with neutral balsam for examination. Photographs were taken (BX51 microscopic/Digital Camera System; Olympus) for study comparison.

The IHC signals were scored as previously described [30]. Briefly, a proportion score was assigned, representing the estimated proportion of positively staining tumor cells (0 = none, 1 = < 1% of cells, 2 = 1% to < 10%, 3 = 1% to 33%, 4 = 33% to 66%, 5 = > 66%). Average estimated intensity of staining in positive cells was assigned an intensity score (0 = none, 1 = weak, 2 = intermediate, 3 = strong). The two parameters were combined, resulting in an overall score (0 or 2 to 8). The scores were classified into three groups: group 1 were slides with a score of 0 (negative expression), group 2 were those scoring 2 to 4 (low expression) and group 3 were those scoring > 5 (high expression). A total of five fields per slide were selected, counted and averaged. Slides were scored by two pathologists (JHW and ACW) who were blinded to the ligand-binding assay results or patient outcome. Consistent with a previous report [31], TERT was found to locate in the nucleus and cytoplasm of cancer cells.

### Statistical analysis

The χ^2 ^test was used to compare gender, ethnicity, tobacco and alcohol use, and family history of first-degree relatives between patients and controls. The unpaired *t*-test was used to analyze differences in mean age and mean smoking level between patients and controls.

Genotype and allele frequencies for the MNS16A polymorphism were determined by gene counting, and departures from Hardy-Weinberg equilibrium were tested using the random-permutation procedure implemented in the Arlequin package [32]. The association between the genotypes and NPC risk was evaluated by multivariate logistic regression analyses. The odds ratio (OR) and 95% confidence interval (CI) were adjusted for age, gender, tobacco and alcohol use, smoking level, ethnicity and family history where appropriate. Potential modification of the effect of MNS16A genotypes on the risk and progression of NPC was assessed for the above confounding factors by addition of interaction terms in the logistic model, and by separate analyses of subgroups of subjects determined by these factors. To assess the probability of a spurious association due to multiple testing, we calculated the false-positive report probability (FPRP) for a polymorphism from an estimated OR and 95% CI, using the method described by Wacholder *et al *[33]. FPRP of < 0. 5 was considered to indicate a robust association.

Differences in the protein levels detected by IHC between the tumor and non-tumor tissue, and between the MNS16A *L *and *S *allele carriers were assessed by a Wilcoxon signed-ranks test and a trend χ^2 ^test, respectively. Logistic regression analysis was also performed for MNS16A genotypes, with the IHC staining scores as the outcome variable.

*P *< 0. 05 was considered significant, and all statistical tests were two-sided. These analyses were performed using SPSS software (version 17. 0, SPSS Inc., Chicago, IL, USA).

## Results

The selected demographic and clinical characteristics of patients with NPC and control subjects were described in detail previously (see Additional file 1, Table S1) [29]. Briefly, the patients were comparable with controls with regard to gender and status of tobacco and alcohol. However, cases were more likely than controls to have an older mean age (*P *= 0. 0011, *t*-test), positive history of NPC in their first-degree biological relatives (χ^2 ^= 15. 96, *P *= 6. 5 × 10^-5^) and non-Han ethnicity (χ^2 ^= 58. 07, *P *= 2. 5 × 10^-14^). An absolute majority of cases (97. 0%) was classified as having poorly differentiated SCC. According to the TNM system, 4. 8%, 46. 2%, 30. 3% and 18. 7% of patients had stage I, II, III and IV disease, respectively.

The genotyping results are shown in Table 1. This case-control study included 855 patients with incident NPC and 1036 control subjects. However, there was failure of 74 samples (57 cases and 17 controls) in the genotyping assay due to DNA quality and/or quantity. Therefore, 798 cases (93. 4%) and 1019 controls (98. 3%) were included in the final analysis for the MNS16A polymorphism. We identified four alleles, of sizs 243, 272, 302 and 333 bp. Only one patient with NPC had the 333 bp allele, and to our knowledge, this is the first report of this allele. Compared with the common 302 bp allele, the short alleles (243 and 272 bp) were not found to be associated with the risk of NPC (all *P *> 0. 05; Table 1). We identified six genotypes in our case-control population of Chinese ancestry (see Additional file 1, Figure S1 and S2, lanes 1 to 6) rather than the seven reported for non-Hispanic whites [25]. The observed genotype frequencies for the MNS16A variant were in Hardy-Weinberg equilibrium (HWE) in both cases (*P *= 0. 58) and controls (*P *= 0. 17). When the commonest genotype (that is, 302/302) was designated as reference group, all the other genotypes were still not associated with the risk of NPC (all *P *> 0. 05; Table 1).

**Table 1:** The genotype and allele frequencies of MNS16A in patients with nasopharyngeal carcinoma and cancer-free controls

MNS16A^a^	Cases, n (%), n = 798^b^	Controls, n (%), n = 1019^b^	OR (95% CI)^c,d^	*P^c^*
**Alleles, bp**				
**302 (reference)**	1520 (95. 2)	1903 (93. 4)	1. 00	
**243**	44 (2. 8)	76 (3. 7)	0. 74 (0. 51 to 1. 08)	0. 12
**272**	31 (1. 9)	59 (2. 9)	0. 65 (0. 42 to 1. 01)	0. 058
**333**	1 (0. 06)	0	-	-
**Genotypes**				
**302/302 (reference)**	724 (90. 7)	891 (87. 4)	1. 00	
**243/302**	41 (5. 2)	65 (6. 4)	0. 80 (0. 53 to 1. 20)	0. 29
**272/302**	30 (3. 8)	56 (5. 5)	0. 64 (0. 41 to 1. 01)	0. 059
**243/243**	1 (0. 1)	4 (0. 4)	0. 36 (0. 04 to 3. 73)	0. 41
**243/272**	1 (0. 1)	3 (0. 3)	0. 33 (0. 04 to 2. 94)	0. 32
**302/333**	1 (0. 1)	0	-	*-*
**Group of genotypes**				
***LL *(reference)**	725 (90. 9)	891 (87. 4)	1. 00	
***SL***	71 (8. 9)	121 (11. 9)	0. 73 (0. 53 to 0. 99)	0. 037
***SS***	2 (0. 2)	7 (0. 7)	0. 35 (0. 07 to 1. 69)	0. 17
***SL *+ *SS***	73 (9. 1)	128 (12. 6)	0. 71 (0. 52 to 0. 96)	0. 025
**Group of alleles**				
***L *(reference)**	1521 (95. 0)	1903 (93. 0)	1. 00	
***S***	75 (5. 0)	135 (7. 0)	0. 86 (0. 78 to 0. 96)	0. 014

**^a^**Owing to genotyping failure, the actual sample size was 798 and 1019 for the cases and controls, respectively.

^b^*L *allele was 302 or 333 bp; *S *allele was 243 or 272 bp.

^c^The odds ratios (ORs) and *P *values were adjusted for age, gender, tobacco and alcohol use, smoking level, ethnicity and family history.

**^d^**Confidence interval.

In accordance with a previous report [21], the 243 bp and 272 bp bands were classified as the short (S) allele, and the 333 bp and 302 bp were classified as the long (L) allele. The MNS16A genotypes were therefore defined as *LL, SL *and *SS*. The frequencies of the *LL, SL *and *SS *genotype were significantly different for cases and controls (χ^2 ^= 6. 06, *P *= 0. 048, degree of freedom (df) = 2), and this difference was mainly caused by the lower frequencies of the *SL *and *SS *genotype in the case group compared with the control group. Based on logistic regression analysis with adjustment for age, gender, tobacco and alcohol use, ethnicity, and family history of NPC, individuals carrying the S alleles (*SL *+ *SS *genotype) had a significantly reduced risk of NPC occurrence compared with those carrying the *LL *genotype (OR = 0. 71, 95% CI = 0. 52 to 0. 96, *P *= 0. 025). This association remained robust given prior probabilities of actual association of 25% (FPRP = 0. 13) and 10% (FPRP = 0. 30), but not with a prior probability of 1% (FPRP = 0. 83) [33]. When cases were limited to those with poorly differentiated SCC who were successfully genotyped (n = 773), the significant association results were more evident (OR = 0. 67, 95% CI = 0. 50 to 0. 90, *P *= 0. 0068). The association between the MNS16A and risk of NPC occurrence were further examined with stratification by age, gender, tobacco and alcohol use, smoking level, ethnicity, and family history (Table 2). Although the decreased risk of NPC associated with the MNS16A *SL *+ *SS *genotypes was more pronounced in subjects who were older (≥ 45 years), male, smokers, drinkers, Han Chinese and with a negative family history, these differences could be attributed to chance (all *P *> 0. 05, test for homogeneity), indicating that these potential confounding factors had no modification effect on the risk of NPC related to MNS16A genotypes.

**Table 2:** Risk of nasopharyngeal carcinoma associated with MNS16A by potential risk factors

Category	*LL*^a,b^	*SL *+ *SS*^a,b^	OR (95% CI)^c,d^	*P*^e^	*P*_homogeneity_^f^
Age, years					0. 55
< 45	331/448	34/60	0. 78 (0. 50 to 1. 22)	0. 19	
≥ 45	394/438	39/68	0. 65 (0. 43 to 0. 98)	0. 051	
Gender					0. 24
Male	525/646	44/88	0. 61 (0. 42 to 0. 90)	0. 0090	
Female	200/245	29/40	0. 90 (0. 54 to 1. 51)	0. 61	
Tobacco use					0. 11
Nonsmoker	502/624	56/85	0. 82 (0. 57 to 1. 18)	0. 28	
Smoker	223/267	17/43	0. 47 (0. 26 to 0. 85)	0. 0090	
Smoking level (pack-years)					0. 34
< 24	635/787	65/109	0. 74 (0. 53 to 1. 03)	0. 071	
≥ 24	90/104	8/19	0. 47 (0. 20 to 1. 13)	0. 080	
Alcohol use					0. 39
Non-drinker	509/630	55/89	0. 76 (0. 53 to 1. 09)	0. 13	
Drinker	217/261	18/39	0. 56 (0. 31 to 1. 02)	0. 032	
Ethnicity					0. 77
Han	541/776	57/112	0. 72 (0. 52 to 1. 02)	0. 064	
Non-Han	184/115	16/16	0. 67 (0. 30 to 1. 50)	0. 67	
Family history^f^					0. 70
Negative	678/862	71/127	0. 69 (0. 51 to 0. 95)	0. 022	
Positive	47/29	2/1	1. 12 (0. 09 to 13. 26)	0. 88	

^a^Owing to genotyping failure, the actual sample size was 798 and 1019 for the cases and controls, respectively.

^b^Number of genotype in cases/number of genotype in controls. *L *allele, *302 *or *333 bp*; *S *allele, *243 *or *272 bp*.

^c^The odds ratios (ORs) and *P *values were calculated by logistic regression with *LL *genotype as the reference group and adjusted for age, gender, tobacco and alcohol status, smoking level, ethnicity and family history where appropriate within the strata.

^d^Confidence interval

^e^For difference in ORs within each stratum.

^f^First-degree relatives

Furthermore, we evaluated the effect of the MNS16A *SL *+ *SS *genotypes on severity of NPC (as measured by the TNM staging system). However, the distributions of the MNS16A genotypes were not significantly different between subgroups with different clinical stages or different TNM classifications (all *P *> 0. 05; Table 3).

**Table 3:** Distributions of genotypes of the MNS16A between the subgroups of cases^a ^with different progression of nasopharyngeal carcinoma (NPC)

NPC stage	***LL***^**b **^**n (%)**	***SL *+ *SS***^**b**^**, n (%)**	**χ**^**2c**^	*P*
Clinical				
I	36 (90. 0)	2 (10. 0)	1. 554	0. 21
II	334 (89. 8)	38 (10. 2)		
III	212 (90. 2)	23 (9. 8)		
IV	143 (93. 5)	10 (6. 5)		
Tumor (T)			3. 314	0. 069
T1	151 (91. 5)	14 (8. 5)		
T2	356 (89. 9)	40 (10. 1)		
T3	139 (89. 7)	16 (10. 3)		
T4	79 (96. 3)	3 (3. 7)		
Node(N)			0. 667	0. 41
N0	148 (89. 2)	18 (10. 8)		
N1	350 (91. 1)	34 (8. 9)		
N2	156 (90. 7)	16 (9. 3)		
N3	71 (93. 4)	5 (6. 6)		
Metastasis(M)			0. 000	1. 00
M0	708 (90. 9)	71 (9. 1)		
M1	17 (89. 5)	2 (10. 5)		

^a^Owing to genotyping failure, the actual sample size of the cases was 798.

^b^*L *allele, 302 or 333 bp; *S *allele, 243 or 272 bp.

^c^Comparisons of genotype distributions between the different subgroups were performed using the χ^2 ^test. The degrees of freedom for the clinical, T and N stages is 3, and for the M stage is 1

In 798 patients with NPC in the Guangxi population, the mean ± SD age at diagnosis was 46. 75 ± 11. 82 years for subjects with the *LL *genotype and 47. 78 ± 13. 58 years for those with the *SL *or *SS *genotype. Therefore, subjects carrying with the protective *S *allele were on average 1. 03 years older at diagnosis than those with the *LL *genotype, although the difference was not significant (*P *= 0. 33, *t*-test).

We further investigated the protein expression of TERT in NPC tissues and non-cancerous nasopharyngeal epithelium tissues by IHC assay (Table 4; see Additional file 1, Table S3). We detected positive expression of TERT in the 34 of 41 NPC tissues (82. 9%), but negative expression in all 13 non-cancerous nasopharyngeal tissues. Furthermore, there was significant association between MNS16A genotypes and expression of TERT in the NPC tissues, with the *SL *genotype carriers having lower TERT expression than the *LL *genotype carriers (*P *= 0. 032, logistic regression analysis; χ^2 ^= 6. 676, *P *= 0. 035).

**Table 4:** Correlation between protein expression levels of telomerase reverse transcriptase (TERT) and MNS16A genotypes by immunohistochemistry

Tissues	**Genotypes**^**a**^	**Expression levels,**^**b**^**n**			***P***^**c**^	
			
		Negative	Low	High	**NPC vs**. non-NPC	***LL *vs**. *SL *+ *SS*
NPC^d ^tissues					4. 78 × 10^-7^	0. 035
	*LL*	5	11	21		
	*SL*	2	2	0		
	*SS*	0	0	0		
Non-cancer^e^						NA^f^
	*LL*	11	0	0		
	*SL*	1	0	0		
	*SS*	1	0	0		

^a^*L *allele, 302 or 333 bp; *S *allele, 243 or 272 bp.

^b^Expression levels were classified into three groups (negative, low and high expression) based on the scores of the immunohistochemistry signals.

^c^The difference of the TERT protein level between the genotypes was assessed by a trend χ^2 ^test. The difference of the protein level between the tumors and non-cancerous nasopharyngeal tissues was assessed by a Wilcoxon signed-ranks test.

^d^Nasopharyngeal cancer

^e^Non-cancerous nasopharyngeal tissues

^f^Not available.

## Discussion

In this study, we assessed the associations of the functional VNTR downstream of the *TERT *gene MNS16A with the risk of occurrence and progression of NPC in the Guangxi population in southern China. No genetic association was found between this polymorphism and the progression of NPC. However, this polymorphism was significantly associated with the onset of NPC, especially in poorly differentiated SCC. This is the first report, to our knowledge, of the genetic association between the *TERT *MNS16A and risk of NPC, confirming the initial hypothesis that the TERT may play a role in the pathogenesis of this malignancy.

In our study population, the *S *allele (*SL *+ *SS *genotype) was over-represented in controls compared with cases. Furthermore, carriers of the *S *alleles tended to have an older age of diagnosis than those of the *LL *genotype, suggesting a protective role of the MNS16A *S *allele against the onset of NPC. It was reported that the region containing MNS16A had some promoter activity that was influenced by the length of the MNS16A tandem-repeats polymorphism [21]. The MNS16A *S *allele, compared with the *L *allele, was associated with higher expression of the *TERT *antisense RNA transcripts. In our study, we further investigated the protein expression of TERT in NPC and non-cancerous nasopharyngeal epithelium tissues by IHC. We found that the *SL *genotype was significantly associated with lower TERT expression than the *LL *genotype (Table 4). Given the role of TERT in the development of NPC, it might be expected that people who carry the MNS16A *S *allele, and thus have more antisense *TERT *RNA and lower TERT protein expression (and thereby lower telomerase activity) over a lifetime, would have a lower susceptibility to developing this disorder. However, the exact functional molecular mechanisms of MNS16A remain unknown. Further studies will be needed to clarify how MNS16A affects antisense RNA transcription and how antisense RNA affects TERT expression.

Recently, the *TERT *MNS16A has been studied in many cancers, but the results are conflicting. Consistent with this study in NPC, one study reported an association of the MNS16A *S *allele with a decreased risk of non-small-cell lung cancer (NSCLC) [21]. By contrast, a very recent study found no association between MNS16A and risk of glioma, glioblastoma and meningioma [27], and some reports have shown a reverse association with the risk allele, with the MNS16A *S *allele conferring an increased risk of breast cancer, glioblastoma and anaplastic gliomas [24,26]. Recently, some reports have questioned the validity of the *S *and *L *combining system, and assessed directly the association of the raw alleles with the diseases. For example, Jin et al. found that the 243 bp allele, but not the 272 bp allele, was associated with the risk of lung cancer in a Korean population [23]. Hofer *et al. *reported that only the 274 bp allele was associated with the risk of colorectal cancer [28]. In this study we found that both the 243 bp and 272 bp alleles were not associated with the risk of NPC. These conflicting results could be attributable to the different ethnicities of study populations and/or different tumorigenesis of different cancers. Additionally, other factors in the studies, such as small sample size or inadequate adjustment for confounding factors, could also cause the inconsistent results. Consequently, additional well-designed case-control studies on a wide spectrum of cancers with ethnically diverse populations are warranted to understand the roles of the MNS16A polymorphism in the etiology of cancers.

We compared the genotype frequencies of the MNS16A polymorphism in populations from other studies based on available published data to identify differences from our own study. We found that the frequencies of the *SS, SL *and *LL *genotype in our controls were 0. 2%, 8. 9% and 90. 9% (Table 1), respectively, similar to those of a Nanjing population of Chinese descent (0. 4%, 9. 8% and 89. 8% in controls, respectively) [24] and a Korean population (0. 2%, 10. 7% and 89. 1% in controls, respectively) [23] described previously. We also found that the frequencies of the MNS16A genotypes were similar for controls of Caucasian descent from America, France, Austria, the UK and Nordic countries, with the *SS, SL *and *LL *genotype frequencies being 9. 2 to 13. 9%, 40. 3 to 47. 2% and 43. 6 to 47. 8%, respectively [21,26-28]. Obviously, the frequencies of the MNS16A genotypes were significantly different from those of populations of Chinese descent (*P *< 0. 05, χ^2 ^test). It remains to be determined whether these differences between ethnic groups influence the risk of developing NPC.

Previous studies have also reported that the *TERT *MNS16A polymorphism was associated with clinical progression in some types of cancer, including clinical stage, tumor stage, lymph-node metastasis and survival time, although the results were contradictory. Wang *et al. *observed a significantly prolonged survival time for patients with glioblastoma with the *SS *genotype compared with the *SL *or *LL *genotype [25]. However, this finding contradicted those from other studies, in which the *LL *genotype was significantly associated with longer survival time in patients with glioblastoma [27] and early-stage (I or II) NSCLC [22], and was associated with decreased risk of axillary lymph-node metastasis in patients with breast cancer [24]. It is also discrepant with the study by Carpentier *et al*., which did not find any survival differences for patients with glioblastoma or anaplastic glioma with different MNS16A genotypes [26]. In patients with NPC, increased telomerase activity and TERT expression was seen more frequently in patients with advanced clinical stage (III to IV) and lymph-node metastasis (N1 to N3) [18,19]. However, in the present study, we did not find any significant associations between the MNS16A polymorphism and the pathological stages of NPC (Table 3). These findings may be due to the different molecular mechanisms of carcinogenesis in different cancers and/or to the different ethnicities of study populations. In addition, these findings could be the result of the limited sample size and statistical power of our study. For example, this study has only a power of 28. 1% (I + II vs. III + IV), 21. 7% (T1 + T2 vs. T3 + T4), 22. 0% (N0 + N1 vs. N2 + N3) and 3. 8% (M0 vs. M1) to detect an OR of 0. 70 for carriers of the *SL *+ *SS *genotypes relative to the carriers of the *LL *genotype. Therefore, we urge that the role of the MNS16A in the progression of NPC be investigated in additional studies with larger sample sizes.

Recently, numerous genome-wide association studies (GWAS) have been carried out to identify associations between cancer risk and single-nucleotide polymorphisms (SNPs). Multiple independent GWAS have all shown the *TERT*-*CLPTM1L *locus on 5p15. 33 to be significantly associated with many different types of cancer, including basal cell carcinoma, glioma, lung, prostate, bladder, testicular germ cell, pancreatic, and cervical cancers [34]. Thus, the evidence that the *TERT *locus contains risk factors for cancer is compelling. Further studies are needed to systematically verify the SNPs at the *TERT *locus, investigate the interactions between these SNPs and MNS16A, and and assess their contributions to the endemics of NPC in the Chinese population.

In reviewing the results of this study, several potential limitations should be kept in mind. First, as this was a hospital-based study, our NPC cases were enroled from the hospitals, and the controls were selected from the community population, thus inherent selection bias cannot be completely excluded. However, through further adjustment and stratification in data analyses, we aimed to minimize the potential confounding effect. Second, several association studies have reported identification of the genes that may relate to the susceptibility to NPC [35-39]; however, most of the results could not be replicated in subsequent studies in other populations. Although we found a significant association between MNS16A and risk of NPC, our initial findings should be independently verified in other populations with a high incidence rate of NPC, such as other southern Chinese, Taiwanese and Singaporeans. Without rigorous replication we cannot exclude the possibility that these findings are due only to chance. Therefore, any association reported in the present study should be interpreted with great caution.

## Conclusions

Our results reveal an association for the first time between the functional tandem repeat polymorphism MNS16A downstream of TERT and susceptibility to NPC. Furthermore, the *S *allele, which is associated with higher expression of the *TERT *antisense RNA and lower expression of the TERT protein, seems to be a genetic protection factor for NPC risk in a Chinese population. If confirmed by other studies, knowledge of genetic factors contributing to the pathogenesis of the NPC as presented here may have implications for the cancer screening and treatment of this disorder in the future.

## Competing interests

The authors declare that they have no competing interests.

## Authors' contributions

YZ conceived and designed the experiments, performed the experiments, analyzed the data, and drafted the manuscript. HZ contributed to the experimental design and data analysis. YZ, ZW, PL and LY contributed to the sample preparation and genotyping. FM, HW, YC contributed to sample collection. YZ contributed to the denatured polyacrylamide gel electrophoresis. GZ and FH conceived and designed the experiments, and drafted and revised the manuscript. All authors read and approved the final manuscript.

## Pre-publication history

The pre-publication history for this paper can be accessed here:

http://www.biomedcentral.com/1741-7015/9/106/prepub

## Supplementary Material

Additional file 1 **Supplemental Table S1, S2 and S3**. Supplemental Figure S1, S2 and S3. Click here for file
