# Supplementary material for: A functional tandem-repeats polymorphism in the downstream of TERT is associated with the risk of nasopharyngeal carcinoma in Chinese population
Source: BMC Med. 2011 Sep 20;9:106. doi: 10.1186/1741-7015-9-106 (PMC3191471; doi:10.1186/1741-7015-9-106)
Supplement: Additional file 1 — Supplemental Table S1, S2 and S3. Supplemental Figure S1, S2 and S3. [file 1741-7015-9-106-S1.DOC]

**Supplementary Tables:**

**Table S1. Selected characteristics of patients with NPC and controls in the Guangxi population.^a^**

| **Category** | **Cases**  **(n = 855)** | **Controls**  **(n = 1036)** | ***P* value** | ***χ^2^*** |
| --- | --- | --- | --- | --- |
| Sex, n (%) |  |  |  |  |
| Male | 616 (72.0) | 749 (72.3) | 0.90 | 0.015 |
| Female | 239 (28.0) | 287 (27.7) |  |  |
| Age, years |  |  |  |  |
| Mean (SD) | 46.5 (11.9) | 44.7 (12.1) | 0.0011 |  |
| ≥ 45, n (%) | 464 (54.3) | 516 (49.8) | 0.053 | 3.74 |
| < 45, n (%) | 391 (45.7) | 520 (50.2) |  |  |
| Smoking status, n (%) |  |  |  |  |
| Smoker | 257 (30.1) | 317 (30.6) | 0.80 | 0.065 |
| Nonsmoker | 598 (69.9) | 719 (69.4) |  |  |
| Smoking level, pack-years |  |  |  |  |
| Mean (SD) | 22.9 (12.9) | 24.3 (18.4) | 0.30 |  |
| ≥ 24, n (%) | 151 (58.8) | 189 (59.6) | 0.83 | 0.044 |
| < 24, n (%) | 106 (41.2) | 128 (40.4) |  |  |
| Drinking status, n (%) |  |  |  |  |
| Drinker | 256 (29.9) | 302 (29.2) | 0.71 | 0.14 |
| Nondrinker | 599 (70.1) | 734 (70.8) |  |  |
| Ethnicity, n (%) |  |  |  |  |
| Han | 628 (73.5) | 904 (87.3) | 2.5 × 10^-14^ | 58.07 |
| Non-Han^b^ | 227 (26.5) | 132 (12.7) |  |  |
| First-family history, n (%) |  |  |  |  |
| Negative | 797 (93.2) | 1006 (97.1) | 6.5 × 10^-5^ | 15.96 |
| Positive | 58 (6.8) | 30 (2.9) |  |  |
| Histological type, n (%) |  |  |  |  |
| Poorly differentiated squamous cell carcinoma | 829 (97.0) |  |  |  |
| Others^c^ | 26 (3.0) |  |  |  |
| Clinical stage, n (%) |  |  |  |  |
| I | 41 (4.8) |  |  |  |
| II | 395 (46.2) |  |  |  |
| III | 259 (30.3) |  |  |  |
| IV | 160 (18.7) |  |  |  |
| Local tumor invasion (T classification), n (%) |  |  |  |  |
| T1 | 170 (19.9) |  |  |  |
| T2 | 424 (49.6) |  |  |  |
| T3 | 174 (20.4) |  |  |  |
| T4 | 87 (10.1) |  |  |  |
| Lymph node involvement (N classification), n (%) |  |  |  |  |
| N0 | 178 (20.8) |  |  |  |
| N1 | 414 (48.4) |  |  |  |
| N2 | 185 (21.6) |  |  |  |
| N3 | 78 (9.2) |  |  |  |
| Distance metastasis (M classification), n (%) |  |  |  |  |
| M0 | 835 (97.7) |  |  |  |
| M1 | 20 (2.3) |  |  |  |

SD, standard deviation; NPC, nasopharyngeal carcinoma. Comparisons of sex, smoker, drinker, ethnicity, and first-family history distributions between patients and controls were performed by use of the *χ^2^* test. Differences of mean age and mean smoking level between patients and controls were analyzed by use of an unpaired *t* test.

^a^Of the 855 cases and 1036 controls involved in the present study, some were derived from our previous study [1] and others were newly recruited [2].

^b^In cases, non-Han includes Zhuang (n = 211), Dong (n = 1), Hui (n = 1), Miao (n=1), Mulao (n = 3) and Yao (n = 10) ethnicity; in controls, non-Han includes Zhuang (n = 132) ethnicity.

^c^Others include vesicular nucleus cell carcinoma (n = 14), poorly differentiated adenocarcinoma (n = 4), and moderate differentiated squamous cell carcinoma (n =5); and undifferentiated cancer (n = 3).

**Table S2. Selected characteristics of patients with NPC and controls for the immunohistochemical staining analysis**.

| **Category** | **Cases**  **(n = 41)** | **Controls**  **(n = 13)** |
| --- | --- | --- |
| Sex, n (%) |  |  |
| Male | 23 (56.1) | 10 (76.9) |
| Female | 18 (43.9) | 3 (23.1) |
| Age, years |  |  |
| Mean (SD) | 44.3 (11.8) | 36.8 (12.5) |
| ≥ 45, n (%) | 15 (36.6) | 3 (23.1) |
| < 45, n (%) | 26 (63.4) | 10 (76.9) |
| Smoking status, n (%) |  |  |
| Smoker | 13 (31.7) | 1 (92.3) |
| Nonsmoker | 28 (68.3) | 12 (7.7) |
| Smoking level, pack-years |  |  |
| Mean (SD) | 7.65 (13.0) | 3.18 (11.1) |
| ≥ 24, n (%) | 7 (17.1) | 1 (100) |
| < 24, n (%) | 34 (82.9) | 12 (0) |
| Drinking status, n (%) |  |  |
| Drinker | 7 (17.1) | 0 (0) |
| Nondrinker | 34 (82.9) | 13 (100) |
| Ethnicity, n (%) |  |  |
| Han | 36 (87.8) | 13 (100) |
| Non-Han | 5 (12.2) | 0 (0) |
| First-family history, n (%) |  |  |
| Negative | 41 (100) | 13 (100) |
| Positive | 0 (0) | 0 (0) |
| Histological type, n (%) |  |  |
| Poorly differentiated squamous cell carcinoma | 41 (100) |  |
| Others | 0 (0) |  |
| Clinical stage, n (%) |  |  |
| I | 0 (0) |  |
| II | 34 (82.9) |  |
| III | 4 (9.75) |  |
| IV | 3 (7.31) |  |
| Local tumor invasion (T classification), n (%) |  |  |
| T1 | 1 (2.43) |  |
| T2 | 27 (65.9) |  |
| T3 | 10 (24.4) |  |
| T4 | 3 (7.31) |  |
| Lymph node involvement (N classification), n (%) |  |  |
| N0 | 3 (7.31) |  |
| N1 | 27 (65.9) |  |
| N2 | 11 (26.7) |  |
| N3 | 0 (0) |  |
| Distance metastasis (M classification), n (%) |  |  |
| M0 | 40 (97.6) |  |
| M1 | 1 (2.43) |  |

SD, standard deviation; NPC, nasopharyngeal carcinoma. The histological type of all tumor tissues was poorly differentiated squamous cell carcinoma. The histological type of all tissues in the controls was chronic inflammation.

**Table S3.** Correlation between protein expression levels of TERT and MNS16A genotypes by IHC assay.

| **Tissues** | **Genotypes ^a^** | **IHC staining scores, n** | | | | | | | | ***P*** | |
| --- | --- | --- | --- | --- | --- | --- | --- | --- | --- | --- | --- |
|  |  | **0** | **2** | **3** | **4** | **5** | **6** | **7** | **8** | **NPC vs.**  **non-NPC** | ***LL* vs.**  ***SL* + *SS*** |
| NPC tissues |  |  |  |  |  |  |  |  |  | 3.68 × 10^-6^ | 0.032 |
|  | *LL* | 5 | 0 | 2 | 9 | 5 | 10 | 5 | 1 |  |  |
|  | *SL* | 2 | 0 | 0 | 2 | 0 | 0 | 0 | 0 |  |  |
|  | *SS* | 0 | 0 | 0 | 0 | 0 | 0 | 0 | 0 |  |  |
| non-cancer nasopharyngeal tissues |  |  |  |  |  |  |  |  |  |  | NA |
|  | *LL* | 11 | 0 | 0 | 0 | 0 | 0 | 0 | 0 |  |  |
|  | *SL* | 1 | 0 | 0 | 0 | 0 | 0 | 0 | 0 |  |  |
|  | *SS* | 1 | 0 | 0 | 0 | 0 | 0 | 0 | 0 |  |  |

IHC, immunohistochemistry. NA, not available. The IHC signals were scored as 0 or 2 - 8. The difference of the TERT protein level between the genotypes was assessed by logistic regression analysis. The difference of the protein level between the tumors and non-cancer nasopharyngeal tissues was assessed by a Wilcox test.

^a^*L* allele, *302* or *333* *bp*; *S* allele, *243* or *272* *bp*.

**Supplementary Figures:**


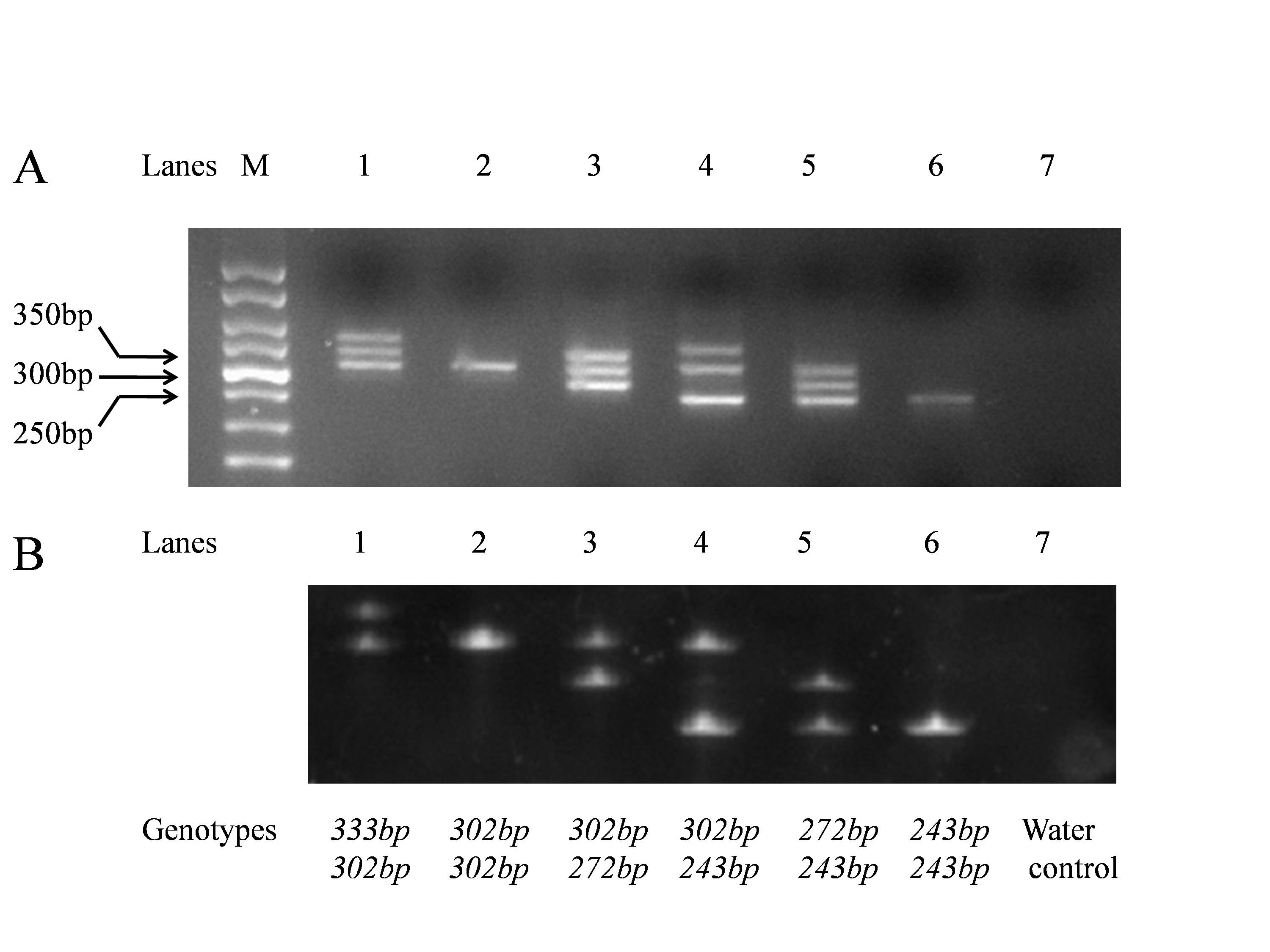
**Figure S1.** (A) MNS16A genotypes were performed by the PCR-based assays (electrophoresis in 2.5% agarose gel). M: 50 base pairs (bp) DNA ladder. Genotype patterns from lane 1 to 6: *333 bp/302 bp, 302 bp/302 bp, 302 bp/272 bp, 302 bp/243 bp, 272 bp/243 bp and 243 bp/243 bp*, respectively. Lane 7: negative water control. Extra one band was located on the top of two bands of every heterozygote (lanes 1, 3, 4 and 5) and resulted from conformation change of the heterozygote with different length of alleles. (B) Denatured PCR products of every MNS16A genotypes, which is from the same sample as the (A), were visualized on an 8% denatured polyacrylamide gel. The order of the genotype patterns was the same as (A).


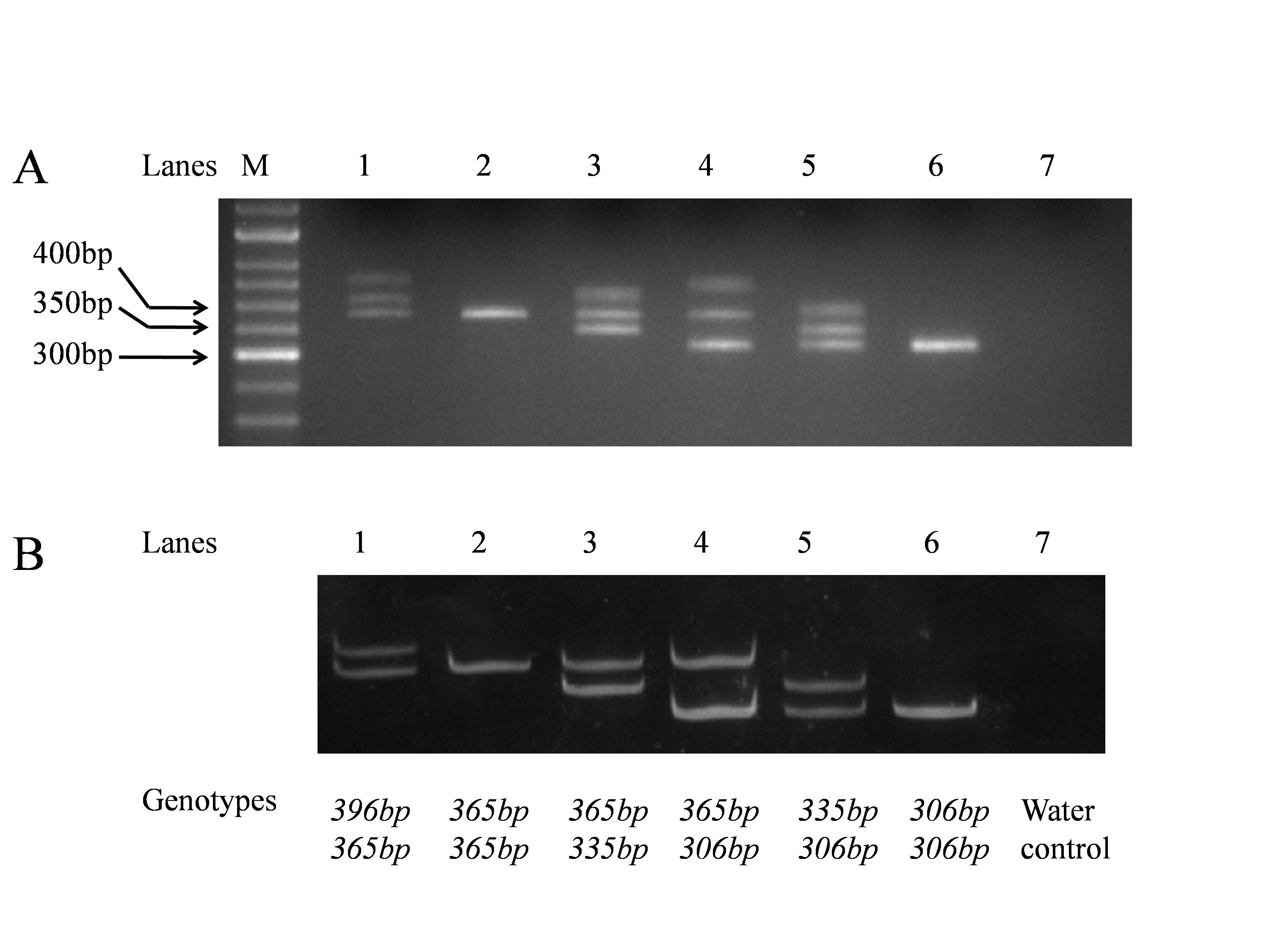


**Figure S2.** (A) MNS16A genotypes of the same individuals as those in Figure S1, were performed by the PCR-based assays (electrophoresis in 2.5% agarose gel) using new primers set we designed. M: 50 bp DNA ladder. Genotype patterns from lane 1 to 6: *396 bp/365 bp, 365 bp/365 bp, 365 bp/335 bp, 365 bp/306 bp, 335 bp/306 bp and 306 bp/306 bp*, respectively. Lane 7: negative water control. Extra one band was located on the top of two bands of every heterozygote (lanes 1, 3, 4 and 5). (B) Denatured PCR products of every MNS16A genotypes, which is from the same sample as the (A), were visualized on an 8% denatured polyacrylamide gel. The order of the genotype patterns was the same as (A).


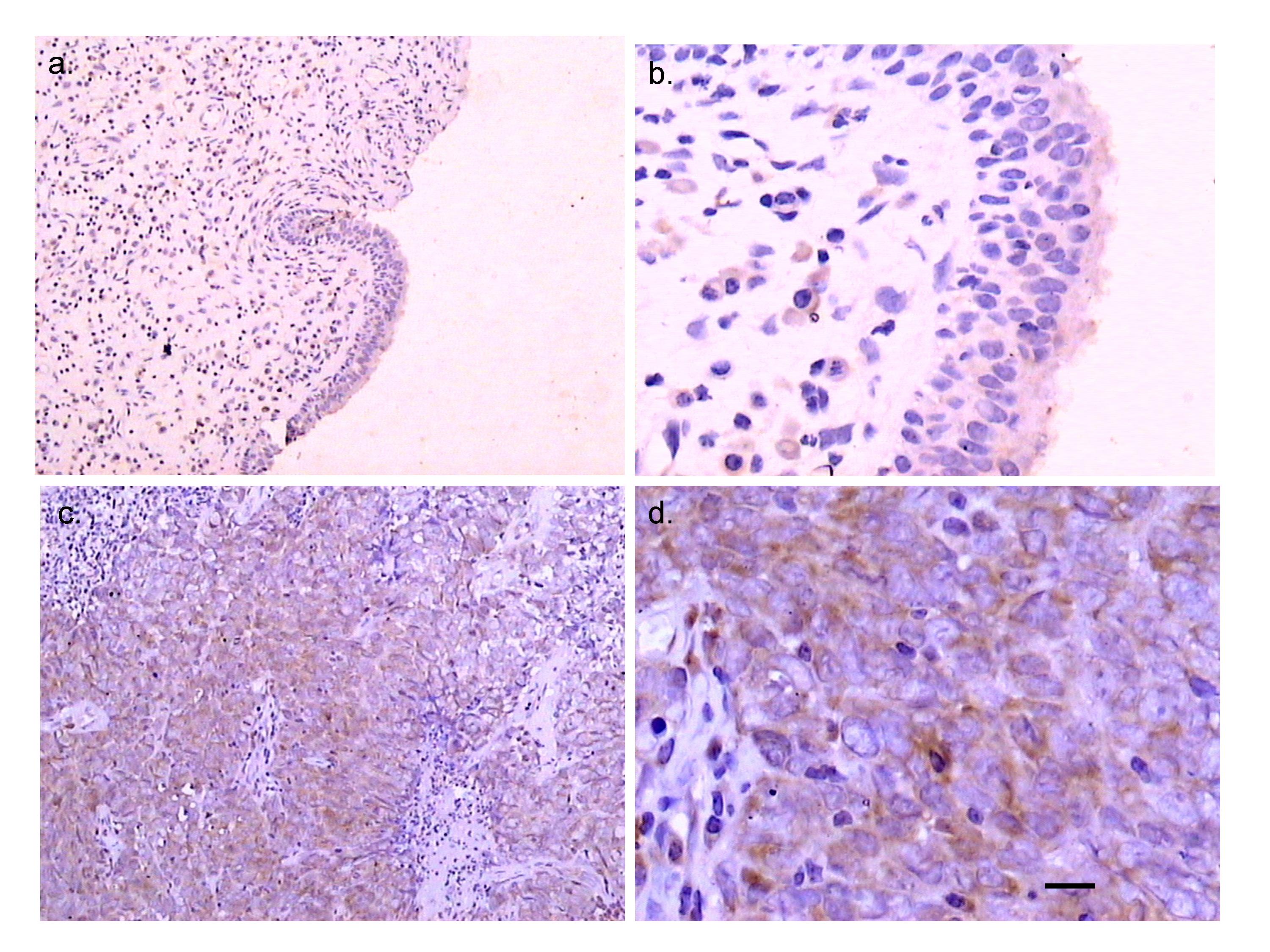
 **Figure S3.** Protein expression of TERT by immunohistochemical staining in representative NPC tissues and non-tumor nasopharyngeal tissues. Panels a and b: adjacent non-tumor tissues; Panels c and d: tumor tissues. The scale bar represents 200 μm in panel a and c, and 50 μm in panels b and d.

**References**

1. Zhou G, Zhai Y, Cui Y, Qiu W, Yang H, Zhang X, Dong X, He Y, Yao K, Zhang H, Peng Y, Yuan X, Zhi L, He F: **Functional polymorphisms and haplotypes in the promoter of the MMP2 gene are associated with risk of nasopharyngeal carcinoma.** *Hum Mutat* 2007, **28:**1091-1097.

2. Ma F, Zhang H, Zhai Y, Huang W, Zhao C, Ou S, Zhou H, Yuan W, Wang Z, Wang H, Yue W, Yu L, Li P, Xia X, Cai M, Zhang Y, Cui Y, He F, Ma Y, Zhou G: **Functional Polymorphism -31C/G in the Promoter of BIRC5 Gene and Risk of Nasopharyngeal Carcinoma among Chinese.** *PLoS One* 2011, **6:**e16748.
